# Sodium dipotassium citrate, NaK_2_C_6_H_5_O_7_

**DOI:** 10.1107/S2056989016002966

**Published:** 2016-02-24

**Authors:** Alagappa Rammohan, James A. Kaduk

**Affiliations:** a Atlantic International University, Honolulu HI , USA; b Illinois Institute of Technology, Chicago IL , USA

**Keywords:** powder diffraction, density functional theory, citrate, sodium, potassium

## Abstract

The crystal structure of sodium dipotassium citrate, Na^+^·2K^+^·C_6_H_5_O_7_^3−^, has been solved and refined using laboratory X-ray powder diffraction data, and optimized using density functional techniques. The Na^+^ and one of the K^+^ cations are six-coordinate, with bond-valence sums of 1.13 and 0.92 valence units, respectively, while another crystallographically independent K^+^ cation is seven-coordinate with a bond-valence sum of 1.20. The [KO_6_] and [KO_7_] polyhedra share edges and corners to form layers perpendicular to the *b* axis. The distorted [NaO_6_] octa­hedra share edges to form chains along the *a* axis. The result is a three-dimensional network. The only O—H⋯O hydrogen bond is an intra­molecular one between the hy­droxy group and a terminal carboxyl­ate group.

## Abstract 2

The crystal structure of sodium dipotassium citrate has been solved and refined using laboratory X-ray powder diffraction data, and optimized using density functional techniques. The Na and K cation coordination spheres share corners and edges to form a three-dimensional network.

## Chemical context   

**Figure 1:**
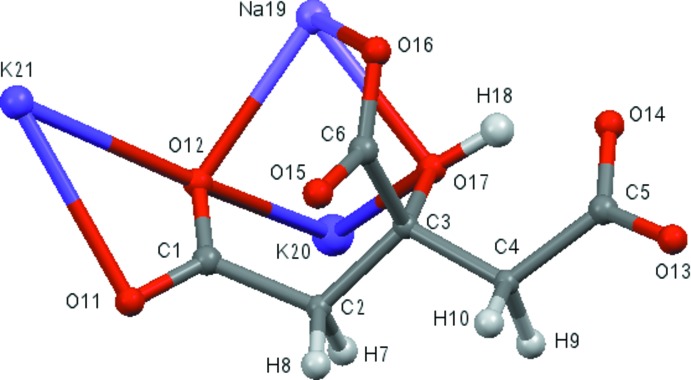
The content of asymmetric unit of the title compound showing the atom numbering and 50% probability displacement spheroids.

We have carried out a systematic study of the crystal structures of Group 1 (alkali metal) citrate salts to understand the anion’s conformational flexibility, ionization, coordination tendencies, and hydrogen bonding. Most of the new structures were solved using powder diffraction data (laboratory and/or synchrotron), but single crystals were used where available. The general trends and conclusions about the 16 new compounds and 12 previously characterized structures are being reported separately (Rammohan & Kaduk, 2016*a* ▸). The initial study considered salts containing one type of Group 1 cations. This compound (Fig. 1 ▸) represents an extension of the study to salts containing more than one alkali metal cation. The structure of related sodium potassium hydrogen citrate has been published recently (Rammohan & Kaduk, 2016*b* ▸).
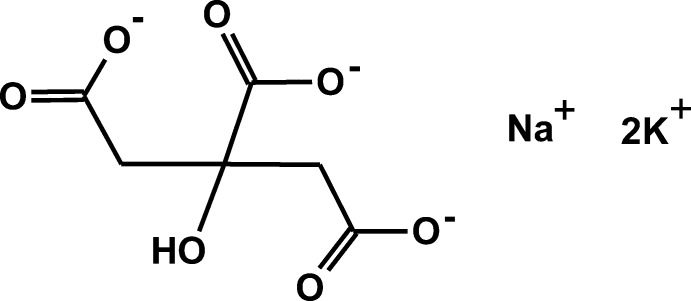


## Structural commentary   

**Figure 2:**
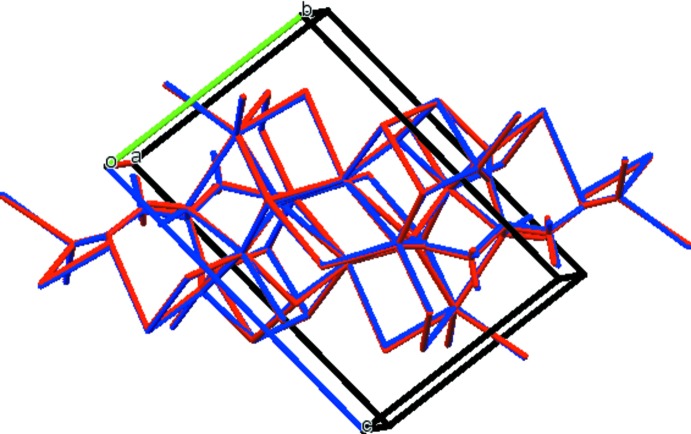
Comparison of the refined and optimized structures of sodium dipotassium citrate. The refined structure is in red, and the DFT-optimized structure is in blue.

The root-mean-square deviation of the non-hydrogen atoms in the refined and optimized structures is only 0.069 Å. The excellent agreement between the structures (Fig. 2 ▸) is strong evidence that the experimental structure is correct (van de Streek & Neumann, 2014 ▸). This discussion uses the DFT-optimized structure. All of the bond lengths and torsion angles, and most of the bond angles fall within the normal ranges indicated by a *Mercury Mogul* Geometry Check (Macrae *et al.*, 2008 ▸). Only the O17—C3—C4 [observed = 115.4 (4), optimized = 109.3, normal = 110.6 (3)°, Z-score = 4.9] and O17—C3—C6 [observed = 109.0 (3), optimized = 111.4, normal = 105.4 (6)°, Z-score = 10.5] angles are flagged as unusual. Part of the reason for the high Z-scores is the exceptionally low standard uncertainties on the normal values. The hy­droxy group O17–H18 bridges Na19 and K20, so a small distortion from the normal geometry may be expected. The citrate anion occurs in the *trans,trans*-conformation (about C2—C3 and C3—C4), which is one of the two low-energy conformations of an isolated citrate. The central carboxyl­ate group and the hy­droxy group occur in the normal planar arrangement. The citrate chelates to Na19 through the terminal carboxyl­ate oxygen O12, the central carboxyl­ate oxygen O17, and the hy­droxy oxygen O17. The citrate chelates to K20 through the terminal carboxyl­ate oxygen O12 and the hy­droxy oxygen O17. One terminal carboxyl­ate group (C1/O11/O12) chelates to K21. Na19 is six-coordinate (distorted octa­hedral), with a bond-valence sum of 1.13 valence units (v.u.). K20 is also six-coordinate with a bond-valence sum of 0.92 v.u.; K21 is seven-coordinate, with a bond-valence sum of 1.20 v.u. Na19 and K21 are thus slightly crowded, while K20 is slightly underbonded. The metal–oxygen bonding is ionic, based on the cation charges and Mulliken overlap populations.

## Supra­molecular features   

**Figure 3:**
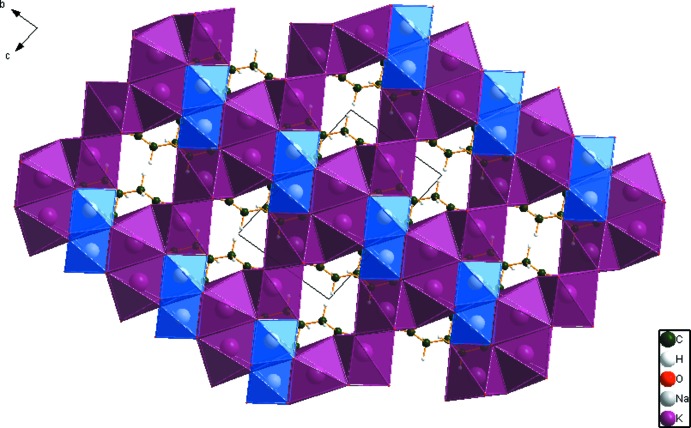
Crystal structure of NaK_2_C_6_H_5_O_7_, viewed approximately down the *a* axis.

In the crystal structure (Fig. 3 ▸), the [KO_6_] and [KO_7_] polyhedra share edges and corners to form layers perpendicular to the *b* axis. The distorted [NaO_6_] octa­hedra share edges to form chains along the *a* axis. The result is a three-dimensional network. The only O—H⋯O hydrogen bond is an intra­molecular one, O17—H18⋯O14 (Table 1 ▸), between the hy­droxy group and a terminal carboxyl­ate. Two inter­molecular C—H⋯O hydrogen bonds also apparently contribute to the crystal energy.

## Database survey   

Details of the comprehensive literature search for citrate structures are presented in Rammohan & Kaduk (2016*a* ▸). A reduced cell search in the Cambridge Structural Database (Groom & Allen, 2014 ▸) (increasing the default tolerance from 1.5 to 2.0%, to account for the differences between ambient and low-temperature lattice parameters) yielded 25 hits, but limiting the chemistry to C, H, O, Na, and K only resulted in no hits. The powder pattern matched no entry in the Powder Diffraction File (ICDD, 2015 ▸).

## Synthesis and crystallization   

2.0764 g (10.0 mmol) H_3_C_6_H_5_O_7_(H_2_O) was dissolved in 20 ml deionized water. 0.5365 g Na_2_CO_3_ (10.0 mmol Na, Sigma–Aldrich) and 1.3824 g K_2_CO_3_ (20.0 mmol K, Sigma–Aldrich) were added to the citric acid solution slowly with stirring. The resulting clear colorless colution was evaporated to dryness in a 393 K oven.

## Refinement details   

**Figure 4:**
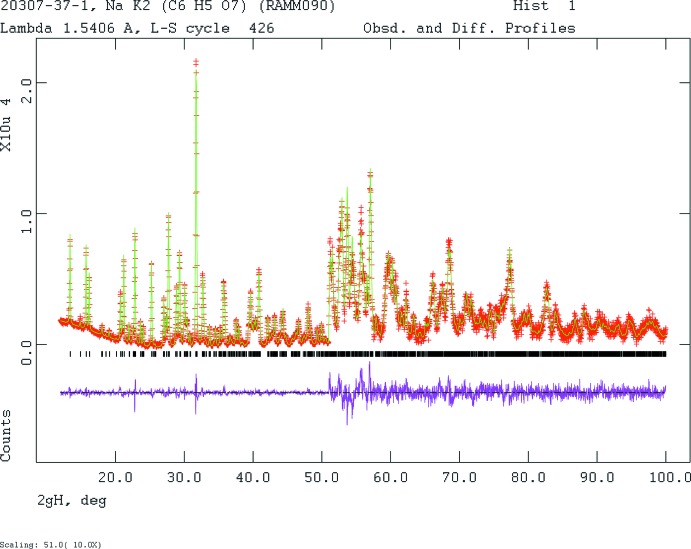
Rietveld plot for the refinement of NaK_2_C_6_H_5_O_7_. The red crosses represent the observed data points, and the green line is the calculated pattern. The magenta curve is the difference pattern, plotted at the same scale as the other patterns. The vertical scale has been multiplied by a factor of 10 for 2θ > 51.0°. The row of black tick marks indicates the Bragg reflection positions for the phase.

Crystal data, data collection and structure refinement details are summarized in Table 2 ▸. The powder pattern (Fig. 4 ▸) was indexed using *Jade 9.5* (MDI, 2012 ▸), which yielded a primitive triclinic unit cell with two formula units and with the lattice parameters as given in Table 2 ▸. Pseudovoigt profile coefficients were as parameterized in Thompson *et al.* (1987 ▸), and the asymmetry correction of Finger *et al.* (1994 ▸) was applied and microstrain broadening by Stephens (1999 ▸). The structure was solved with *FOX* (Favre-Nicolin & Černý, 2002 ▸) using a citrate, Na, and two K as fragments. One of the 10 solutions (2 × 10^6^ moves, with a bump penalty with weighting factor = 50) yielded a much lower cost function than the others. All C—C and C—O bond lengths were restrained, as were all bond angles. The hydrogen atoms were included at fixed positions, which were re-calculated during the course of the refinement. The *U*_iso_ parameters of C2, C3, and C4 were constrained to be equal, and those of H7, H8, H9, and H10 were constrained to be 1.3 times that of these carbon atoms. The *U*_iso_ parameters of C1, C5, C6, and the oxygen atoms were constrained to be equal, and that of H18 was constrained to be 1.3 times this value.

The Bravais–Friedel–Donnay–Harker (Bravais, 1866 ▸; Friedel, 1907 ▸; Donnay & Harker, 1937 ▸) morphology suggests that we might expect platy morphology for sodium dipotas­sium citrate, with {001} as the principal faces. A 2nd-order spherical harmonic preferred orientation model was included in the refinement. The texture index was only 1.006, indicating that preferred orientation was not significant in this rotated flat-plate specimen. The powder pattern is included in the Powder Diffraction File as entry 00-065-1254.

### Density functional geometry optimization   

A density functional geometry optimization (fixed experimental unit cell) was carried out using *CRYSTAL09* (Dovesi *et al.*, 2005 ▸). The basis sets for the H, C, and O atoms were those of Gatti *et al.* (1994 ▸), the basis sets for Na and K were those of Dovesi *et al.* (1991 ▸). The calculation used 8 k-points and the B3LYP functional, and took about 41 h on a 2.8 GHz PC. The *U*_iso_ parameters from the Rietveld refinement were assigned to the optimized fractional coordinates.

## Supplementary Material

Crystal structure: contains datablock(s) RAMM090_publ, ramm090_DFT. DOI: 10.1107/S2056989016002966/cv5503sup1.cif

Supporting information file. DOI: 10.1107/S2056989016002966/cv5503RAMM090_publsup2.cml

CCDC references: 1454587, 1454586

Additional supporting information:  crystallographic information; 3D view; checkCIF report

## Figures and Tables

**Table 1:** Hydrogen-bond geometry (Å, °) for the DFT-optimized structure[Chem scheme1]

*D*—H⋯*A*	*D*—H	H⋯*A*	*D*⋯*A*	*D*—H⋯*A*
O17—H18⋯O14	0.989	1.721	2.614	148.2
C2—H7⋯O13	1.095	2.480	3.448	165.8
C2—H8⋯O17	1.089	2.382	3.513	149.0

**Table 2:** Experimental details

	Powder data
Crystal data	
Chemical formula	Na^+^·2K^+^·C_6_H_5_O_7_^3−^
*M*_r_	290.29
Crystal system, space group	Triclinic, *P* 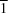
Temperature (K)	300
*a*, *b*, *c* (Å)	5.51284 (12), 7.62583 (13), 11.37121 (14)
α, β, γ (°)	83.4276 (17), 88.991 (2), 84.3488 (16)
*V* (Å^3^)	472.59 (1)
*Z*	2
Radiation type	*K*α_1_, *K*α_2_, λ = 1.540629, 1.544451 Å
Specimen shape, size (mm)	Flat sheet, 24 × 24

Data collection	
Diffractometer	Bruker D2 Phaser
Specimen mounting	Standard holder
Data collection mode	Reflection
Scan method	Step
2θ values (°)	2θ_min_ = 4.908 2θ_max_ = 99.914 2θ_step_ = 0.020

Refinement	
*R* factors and goodness of fit	*R*_p_ = 0.030, *R*_wp_ = 0.039, *R*_exp_ = 0.023, *R*(*F* ^2^) = 0.042, χ^2^ = 3.062
No. of parameters	87
No. of restraints	29
H-atom treatment	Only H-atom displacement parameters refined

The same symmetry and lattice parameters were used for the DFT calculation. Computer programs: *DIFFRAC* (Bruker, 2009 ▸), *PowDLL* (Kourkoumelis, 2013 ▸), *FOX* (Favre-Nicolin & Černý, 2002 ▸), *GSAS* (Larson & Von Dreele, 2004 ▸), *EXPGUI* (Toby, 2001 ▸), *DIAMOND* (Crystal Impact, 2015 ▸) and *publCIF* (Westrip, 2010 ▸).

## supplementary crystallographic information

### Crystal data

**Table:**

NaK_2_C_6_H_5_O_7_	α = 83.4276°
*M**_r_* = 290.27	β = 88.9910°
Triclinic, *P*1	γ = 84.3488°
Hall symbol: -P 1	*V* = 472.59 Å^3^
*a* = 5.5128 Å	*Z* = 2
*b* = 7.6258 Å	*T* = 300 K
*c* = 11.3712 Å	

### Data collection

**Table:**

Density functional calculation	

### Fractional atomic coordinates and isotropic or equivalent isotropic displacement parameters (Å^2^)

**Table:**

	*x*	*y*	*z*	*U*_iso_*/*U*_eq_	
C1	0.23528	0.68012	0.69007	0.01520*	
C2	0.20379	0.79317	0.79461	0.01140*	
C3	0.32923	0.96572	0.77770	0.01140*	
C4	0.23685	1.08853	0.87187	0.01140*	
C5	0.37097	1.25602	0.87268	0.01520*	
C6	0.26511	1.06433	0.65294	0.01520*	
H7	0.27614	0.71264	0.87452	0.01480*	
H8	0.01064	0.82948	0.80961	0.01480*	
H9	0.25919	1.01393	0.96018	0.01480*	
H10	0.04244	1.12921	0.86015	0.01480*	
O11	0.04358	0.63007	0.64853	0.01520*	
O12	0.44832	0.64571	0.65042	0.01520*	
O13	0.25846	1.38757	0.91438	0.01520*	
O14	0.59064	1.25043	0.83476	0.01520*	
O15	0.04336	1.07764	0.62268	0.01520*	
O16	0.43463	1.12385	0.58916	0.01520*	
O17	0.58637	0.92180	0.79054	0.01520*	
H18	0.64696	1.03836	0.79618	0.01980*	
Na19	0.75225	0.87197	0.59720	0.02020*	
K20	0.76524	0.55149	0.86072	0.02970*	
K21	0.25613	0.63139	0.41533	0.01970*	

### Bond lengths (Å)

**Table:**

C1—C2	1.544	C4—C5	1.539
C1—O11	1.274	C4—H9	1.099
C1—O12	1.265	C4—H10	1.092
C2—C3	1.537	C5—O13	1.262
C2—H7	1.095	C5—O14	1.277
C2—H8	1.089	C6—O15	1.267
C3—C4	1.549	C6—O16	1.261
C3—C6	1.557	O17—H18	0.989
C3—O17	1.430		

### Hydrogen-bond geometry (Å, º)

**Table:**

*D*—H···*A*	*D*—H	H···*A*	*D*···*A*	*D*—H···*A*
O17—H18···O14	0.989	1.721	2.614	148.2
C2—H7···O13	1.095	2.480	3.448	165.8
C2—H8···O17	1.089	2.382	3.513	149.0
